# Slow Gait Speed and Rapid Renal Function Decline Are Risk Factors for Postoperative Delirium after Urological Surgery

**DOI:** 10.1371/journal.pone.0153961

**Published:** 2016-05-04

**Authors:** Tendo Sato, Shingo Hatakeyama, Teppei Okamoto, Hayato Yamamoto, Shogo Hosogoe, Yuki Tobisawa, Tohru Yoneyama, Eiji Hashiba, Takahiro Yoneyama, Yasuhiro Hashimoto, Takuya Koie, Kazuyoshi Hirota, Chikara Ohyama

**Affiliations:** 1 Department of Urology, Hirosaki University Graduate School of Medicine, Hirosaki, Japan; 2 Department of Advanced Transplant and Regenerative Medicine, Hirosaki University Graduate School of Medicine, Hirosaki, Japan; 3 Department of Anesthesiology, Hirosaki University Graduate School of Medicine, Hirosaki, Japan; University of British Columbia, CANADA

## Abstract

**Objectives:**

The aim of this study was to identify risk factors associated with postoperative delirium in patients undergoing urological surgery.

**Methods:**

We prospectively evaluated pre- and postoperative risk factors for postoperative delirium in consecutive 215 patients who received urological surgery between August 2013 and November 2014. Preoperative factors included patient demographics, comorbidities, and frailty assessment. Frailty was measured by handgrip strength, fatigue scale of depression, fall risk assessment, and gait speed (the timed Get-up and Go test). Postoperative factors included types of anesthesia, surgical procedure, renal function and serum albumin decline, blood loss, surgery time, highest body temperature, and complications. Uni- and multivariate logistic regression analyses were performed to assess pre- and postoperative predictors for the development of postoperative delirium.

**Results:**

Median age of this cohort was 67 years. Ten patients (4.7%) experienced postoperative delirium. These patients were significantly older, had weak handgrip strength, a higher fall risk assessment score, slow gait speed, and greater renal function decline compared with patients without delirium. Multivariate analysis revealed slow gait speed (>13.0 s) and rapid renal function decline (>30%) were independent risk factors for postoperative delirium.

**Conclusions:**

Slow gait speed and rapid renal function decline after urological surgery are significant factors for postoperative delirium. These data will be helpful for perioperative patient management. This study was registered as a clinical trial: UMIN: R000018809.

## Introduction

Delirium after surgery is a big problem with consequences for patients and healthcare. Postoperative delirium incidences tend to increase in elderly patients[1] and varies from 3.1% to 54.9%.[2–6] The older age is characteristic of aging of organs, increased occurrence of cognitive decline and functional impairment, and these are a large group of patients undergoing urological surgery. Incidence peaks of kidney, urinary bladder and prostate cancers were around 60–80 years of age. Because the development of delirium in surgical patients has been associated with negative outcomes including a higher risk of postoperative mortality, [2, 7, 8] geriatric assessment in older patients with genitourinary cancers, and management of postoperative delirium is an important issue. [9] However, only few studies included very few patients have evaluated postoperative delirium incidences after urological surgery.[3, 4, 10] Postoperative delirium incidences are reported to be 8.8% in general urological surgery,[3] 29% in radical cystectomy,[4] and 21% in transurethral resection of prostate.[10] Identifying postoperative risk assessment is important in preventing delirium but remains challenging due to numerous causes. Several risk factors have been identified, including older age (≥65 years), impaired outcome of cognitive and mental tests, poor nutritional status. However, the incidences of postoperative delirium in urological surgery vary widely because the etiology of delirium is multifactorial. In addition, it is challenging to apply all predictive factors into clinical practice without a well-trained geriatric care team with larger sample size. Therefore, an easy and simple method to evaluate frailty syndrome is required.

In 2004, the American Geriatric Society consensually defined “frailty” as “an excess vulnerability to stressors, with a reduced ability to maintain or regain homeostasis after a destabilizing event.[11] Physical parameters, such as gait speed, are an indicator of functional capacity and frailty syndrome.[12, 13] In recent years, there has been growing interest in measuring patients' gait speed at usual pace to screen for frailty.[14] This screening test is indeed quick, low-cost, and reproducible. Therefore, we hypothesized that physical frailty, including slow gait speed, may be associated with postoperative delirium in patients with urological surgery. The primary aim of the prospective study was to identify pre- and postoperative predictors for developing postoperative delirium after urological surgery. This study was registered as a clinical trial: UMIN: R000018809.

## Methods

We prospectively evaluated frailty patients who admitted to our hospital for urological treatment between August 2013 and November 2014. In this period, 303 patients were admitted in our urological unit. Patients with non-surgical treatments (n = 82) were not included in this study. Of the remaining 221 patients, 2 patients did not undergo surgery due to comorbidities, and we excluded 4 patients due to insufficient data. Finally, we included a consecutive 215 patients (median age, 67 years) who received urological surgery. Delirium was diagnosed based on Diagnostic and Statistical Manual of Mental Disorders–V (DSM-V) criteria [15]. We screened five DSM-V criteria for delirium: A, There is a disturbance in attention and awareness (asking the same questions over and over and/or not be able to have a conversation). B, Delirium develops over a short period of time, typically hours to days. C, There is also another disturbance in cognition, such as in memory, orientation, language, and perception. D, The disturbances in (A) and (C) are not better explained by another pre-existing, established, or evolving neurocognitive disorder (Essential to the diagnosis of delirium is that the patient can respond to “verbal stimulation”). E, There must also be evidence that the delirium is due to a direct physiological consequence of another medical condition, substance intoxication or withdrawal, or exposure to a toxin, or is due to multiple etiologies. If patients met any of these criteria, patients were diagnosed as a delirium. Well-experienced nurses assessed the DSM-V criteria once or twice a day during third postoperative day, and once a day afterwards. In addition, primary doctors investigated delirium at least once a day for all the length of hospital stay.

Inclusion criteria were major and minor surgeries for benign and malignant diseases. In major surgery, we included patients undergoing radical prostatectomy, radical or partial nephrectomy, radical cystectomy, laparoscopic adrenalectomy, renal transplantation, ureterocystoneostomy, retroperitoneal sarcoma resection, and repair of urethra-perineal fistula using a gracilis muscular flap after low anterior resection. Minor surgery included endoscopic transurethral resection of bladder tumor, transurethral cystolithotomy, and high orchiectomy. Exclusion criteria included admission for diseases with severe functional or cognitive impairment (who could not answer functional or cognitive questions), vision disorder or hearing loss, preexisting apparent dementia and cognitive loss, poor general health (ECOG performance status > 2), medical conditions likely to result in death within a few months, or any other reasons because of which patients were unable to perform physical tests or answer the questionnaire on fatigue. In addition, obvious demented people were excluded at the out-patient clinic because these patients were not indicated for urological surgery. These criteria were assessed by well-experienced nurses and primary doctors.

### Ethics Statement

This study was conducted in accordance with the ethical standards of the Declaration of Helsinki and approved by the ethical committee of Hirosaki University Graduate School of Medicine (authorization number: 2014–297). The participants in this study provide their written informed consent.

### Preoperative period

Patients underwent a preoperative evaluation, which included patient characteristics and a frailty assessment, for 1 to 2 weeks before surgery. We assessed a frailty according to the modified Fried criteria,[16] which was focused on physical frailty and the questionnaire of fatigue. Physical frailty was measured by gait speed (the timed Get-up and Go test) and handgrip strength. The timed Get-up and Go test measures, in seconds, the time taken by an individual to stand up from a chair, walk a distance of 3 m, turn, walk back to the chair, and sit down again. The presence of underlying depression and depressive symptoms were assessed using the fatigue scale of the Center for Epidemiologic Studies for Depression (CES-D). Furthermore, fall risk was investigated in this study. We used our original fall risk assessment scale,[17] which is a modified version of the Morse Fall Scale.[18] This fall risk assessment scale included age, past history of falls, vision or hearing disorder, functional disorder, activity, cognition, medication, and egestion. These assessments were performed at the first or second day of admission by several well-experienced nurses. We confirmed interrater reliability of the timed Get-up and Go test, handgrip strength and fall risk assessment.

Routine laboratory investigations were conducted including blood count, serum electrolyte and albumin levels, and liver and renal function tests. Renal function was evaluated using estimated glomerular filtration rate (eGFR), with a modified version of the abbreviated Modification of Diet in Renal Disease Study formula: eGFR mL/min/1.73 m^2^ = 194 × sCr^−1.094^ × age^−0.287^ (×0.739, if female).[19] Nutritional status was evaluated by the Geriatric Nutritional Risk Index {GNRI = [1.489 + albumin (g/L)] + [41.7 × body mass index/22]}, developed as a tool for assessing nutritional risks, with the cut-off value of GNRI at <92.[20, 21]

The choices for anesthesia (general or regional) were decided by one or two senior supervisory doctors in our department, depending on the surgical risk, clinical condition, and comorbidities. The American Society of Anesthesiologists (ASA) classification [22] was used for assessing systemic comorbidity/performance, and it was obtained from the anesthesia chart.

### Anesthetic procedure and intraoperative period

The anesthetic management and postoperative analgesia of patients was consistent and was not modified during this study. All major surgeries were conducted using general anesthesia, induced with remifentanil (0.2–0.5 μg/kg/min), ketamine (0.1–1.0 mg/kg), and propofol (1–2 mg/kg). Tracheal intubation was facilitated with rocuronium bromide (0.6 mg/kg). Anesthesia was maintained with a continuous infusion of remifentanil (0.2–0.5 μg/kg/min), ketamine (0.5–1.0 mg/kg/h), and propofol (4–6 mg/kg/h). Before the end of the surgery, intravenous morphine (5–10 mg) or fentanyl (2–4 μg/kg) was administered as boluses for an opioid rotation. Thereafter, intravenous patient-controlled analgesia using morphine (20–30 mg/day) or fentanyl (400–500 μg/day) with ketamine (20 mg/day) was followed to manage postoperative pain. General or regional anesthesia was also administered for minor surgeries. General anesthesia was similar to that used for open surgeries. Regional spinal anesthesia was induced by injecting 1.6–2.8 mL (body weight × 0.04 mL) of hyperbaric bupivacaine 0.5% into the vertebral space. Moreover, intraoperative data were reviewed, including the type of surgery (major or minor), type of anesthesia (general or regional), and duration and blood loss of surgery.

### Postoperative period

Postoperative data collection included the occurrence of delirium, complications, highest body temperature, renal function and serum albumin decline, and length of hospital stay. Postoperative renal function and serum albumin were evaluated 1 day after surgery. Complications were classified according to the Clavien–Dindo classification[23] and assessed in the postoperative period for up to 30 days.

### Statistical analysis

Statistical analyses were conducted using GraphPad Prism version 5.03 (GraphPad software, Inc. La Jolla, CA, USA) and SPSS software package version 19.0 (SPSS, Chicago, IL, USA) with a *P*-value < 0.05 (two-tailed) considered statistically significant. Quantitative variables were expressed as median, with quartiles 1 and 3 (Q1 and Q3). The between-group difference was statistically compared using the Student’s *t*-test for normal distribution or the Mann–Whitney *U* test for non-normal distribution. Categorical variables were reported as percentages (%) and compared using Fisher’s exact test. To determine predictive factors for postoperative delirium, patients were classified into two groups based on whether postoperative delirium occurred. Optimal cut-offs of age, Get-up and Go test, and fall risk assessment score were calculated by the formula (1 − sensitivity)^2^ + (1 − specificity)^2^, with the help of receiver operator characteristics (ROC) curves.[24]

Here we separately analyzed pre- and postoperative variables. The preoperative risk factors for postoperative delirium were examined by uni- and multivariate logistic regression analyses including the following variables: age (>75 years), sex (male), body mass index (<20 kg/m^2^), medical history of type 2 diabetes, handgrip strength (male <16 kg, female <18 kg), Get-up and Go test (>13 s), CES-D fatigue questionnaire (yes), fall risk assessment (>10 points), nutritional status (GNRI < 92), and ASA status (score 3). For postoperative risk factors, highest body temperature recorded (>38.0°C), postoperative complications (Clavien–Dindo > 1), type of surgery (open), type of anesthesia (general), eGFR decline (>30%), serum albumin decline (>30%), blood loss (>1000 g), and duration of surgery (>3 h). Multivariate logistic regression was used to calculate odds ratios and 95% confidence intervals (CIs) after simultaneously controlling for potential confounders. Patients were categorized according to the number of independent predictors. The predictive accuracy of selected variables for postoperative delirium were evaluated by an area under the curve (AUC) derived from an ROC curve.

## Results

### Characteristics of patients

Of 215 surgical patients, 10 (4.7%) developed postoperative delirium. Median age of the present study was 67 years old (interquartile range 63–75). According to types of surgical procedures, the occurrence of postoperative delirium was highest in nephrectomy and nephroureterectomy (30%), followed by partial nephrectomy 8 (10%), radical prostatectomy (10%), radical cystectomy (10%) and transurethral resection of bladder tumor (10%). All episodes of delirium occurred within 3 days after surgery. Pre- and postoperative variables are listed in Table 1. Details of surgical procedures are shown in Table 2. Median eGFR change between preoperative and the day after surgery in radical prostatectomy, radical cystectomy, radical nephrectomy, partial nephrectomy, nephroureterectomy, other major surgeries (except for renal transplantation), and minor surgeries were 4.0%, -9.0%, -39%, -21%, -23%, 5%, and 1%, respectively. The minimal data set of the present study is available in S1 Dataset.

**Table 1:** Patients’ characteristics.

Preoperative factors	No delirium	With delirium	*P value*
Number of patients, n =	205	10	
Age, years	67 (62, 74)	79 (77, 80)	*< 0*.*001*
Gender (Male / Female), n =	167 / 38	8 / 2	*0*.*908*
Body mass index, kg/m^2^	22.9±2.7	23.1±3.4	*0*.*665*
Type 2 diabetes, n =	17 (8%)	3 (30%)	*0*.*054*
Handgrip strength (Kg)	32 (25, 38)	23 (18, 26)	*0*.*002*
Get-up and Go test (seconds)	9.0 (7.5, 11.0)	15.8 (13.6, 17.3)	*< 0*.*001*
CES-D Fatigue (Yes)	18 (9%)	3 (30%)	*0*.*062*
Fall risk assessment score	5.0 (3.0, 8.0)	11.0 (6.0, 12.5)	*0*.*002*
Nutritional status (GNRI)	104 (98, 110)	104 (101, 108)	*0*.*656*
ASA score	2 (2, 2)	2 (2, 3)	*0*.*151*
Types of disease, n =			
Prostate Cancer	86	1	
Bladder Cancer	53	2	
Renal cell carcinoma	29	4	
Adrenal tumor	10	0	
Renal transplantation	10	0	
Upper tract urothelial carcinoma	7	3	
Others	10	0	
Postoperative factors	No delirium	With delirium	*P value*
Type of anesthesia, n =			*0*.*712*
General	176	9	
Regional (spinal or local)	29	1	
Type of surgical procedure, n =			*0*.*100*
Open	42	5	
Laparoscopic/Robotic	122	4	
Minor	41	1	
Renal function decline, %	2 (-8, 16)	33 (28, 39)	*0*.*002*
Serum albumin decline, %	21 (15, 25)	24 (19, 26)	*0*.*164*
Blood loss, g	40 (5–100)	85 (21, 663)	*0*.*262*
Surgery time, min	159 (112, 188)	145 (131, 180)	*0*.*655*
Highest body temperature, °C	37.5 (37.2, 37.8)	38.0 (37.8, 38.0)	*0*.*191*
Complications (Clavian-Dindo > 1)	29 (14%)	1 (10%)	*1*.*000*
Hospital stay, days	15.0 (11.0, 18.0)	16.0 (12.5, 28.0)	*0*.*446*

Quantitative variables were expressed as median with quartile (Q1 and Q3).

**Table 2:** Types of surgeries. We divided our urological surgery to two groups, major and minor. Surgeries under general anesthesia and local or spinal anesthesia were regarded as major and minor, respectively.

	No delirium	With delirium
1. Major surgeries		
Radical prostatectomy	86 (42%)	1 (20%)
Open	7	0
Robotic	79	1
Radical cystectomy	14 (7%)	1 (10%)
Open	12	1
Robotic	2	0
Radical nephrectomy	28 (14%)	3 (30%)
Open	8	2
Laparoscopic	20	1
Partial nephrectomy	11 (5.4%)	1 (10%)
Open	7	0
Robotic	4	1
Nephroureterectomy	7 (3.4%)	3 (30%)
Open	1	2
Laparoscopic	6	1
Others	18 (9%)	0 (0%)
Open procedures	3	0
Laparoscopic Adrenalectomy	10	0
Renal transplantation	4	0
Laparoscopic lymph node biopsy	1	0
2. Minor surgeries	41 (20%)	1 (10%)
Transurethral resection of bladder tumor	39	1
Orchiectomy	1	0
Transurethral lithotripsy of bladder	1	0

### Comparison of the patients

**Fig 1:**
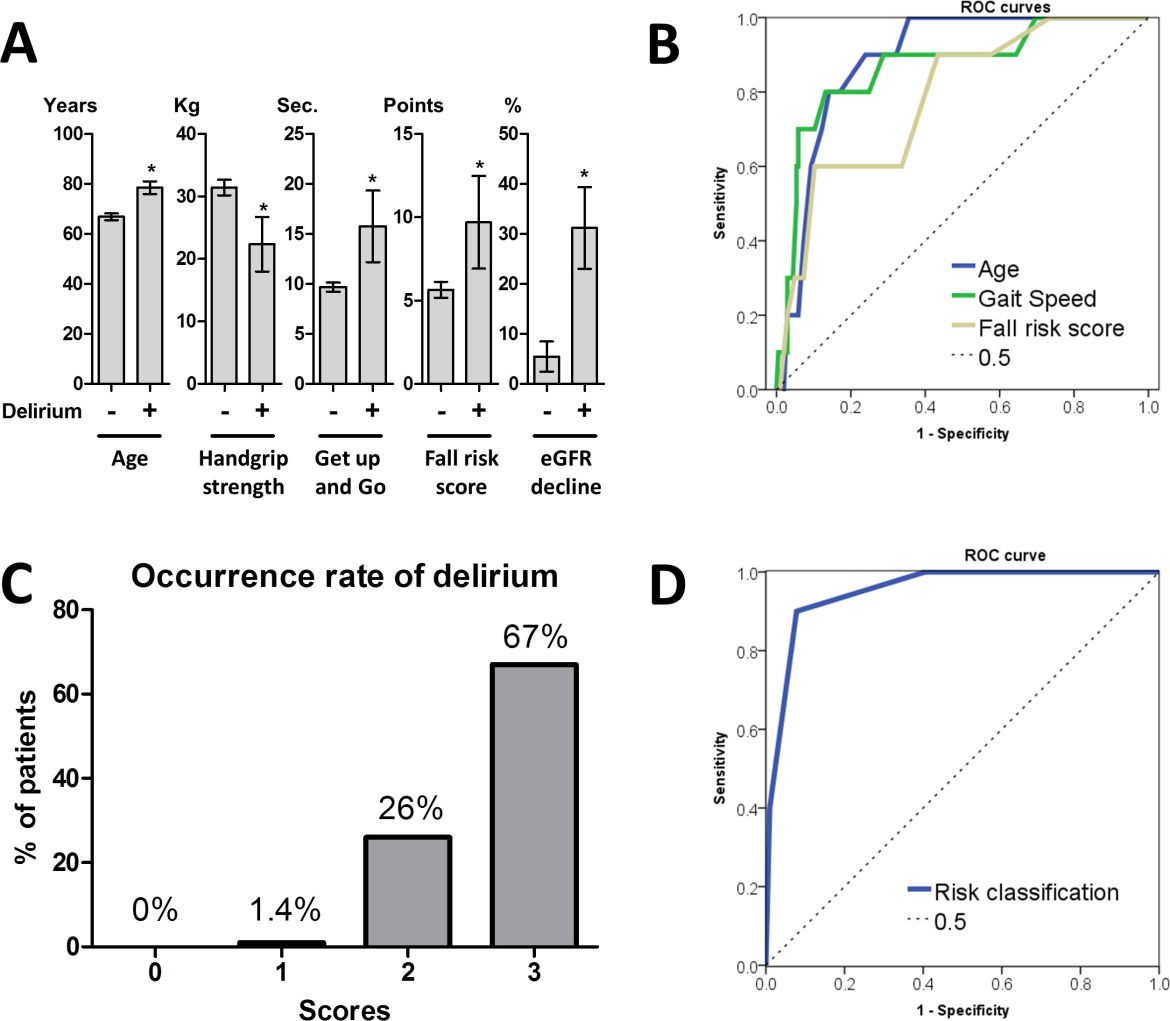
Predictive value of the risk stratification. **A:** Patients characteristics that were significantly different between the groups (*, *P* < 0.01). Error bar demonstrated 95% CI. **B:** ROC curve analysis to determine optimal cut-off values of age, gait speed in Get-up and Go test, and fall risk assessment scores. The AUC values were 0.89 in age, 0.87 in Get-up and Go test, and 0.79 in fall risk assessment score. The optimal cut-off values were age older than 75 years, slower than 13.0 s in the Get-up and Go test, and higher than 10 points in fall risk assessment score. **C:** Patients were categorized according to the number of independent predictors (>75 years old, Get-up and Go > 13.0 s, eGFR decline > 30%) for postoperative delirium (scores 0–3). The occurrence of postoperative delirium was 0% in score 0, 1.4% in score 1, 26% in score 2, and 67% in score 3 (*P* < 0.001). **D:** Predictive accuracy of selected three factors by the ROC curve showed that the AUC value was 0.952 (*P* < 0.001, 95% CI 0.902–1.00).

Patients with delirium were significantly older (*P* < 0.001), had weaker handgrip strength (*P* = 0.002), slower gait speed in the timed Get-up and Go test (*P* < 0.001), and higher fall risk assessment (*P* = 0.002) than patients without delirium in preoperative factors. No patients had experienced previous postoperative delirium in this study. Except for a medical history of type 2 diabetes that showed marginal (*P =* 0.054) differences, there was no difference between patients with or without delirium in gender, body mass index, and nutritional status between the groups. In postoperative factors, no differences were observed between patients with or without delirium, except for rapid eGFR decline (*P =* 0.002; Fig 1A).

Optimal cut-off values of age, gait speed in the Get-up and Go test, and fall risk assessment scores were determined by ROC curves. Age greater than 75 years (AUC = 0.89, *P* < 0.001, 95% CI 0.82–0.95), gait speed slower than 13.0 s (AUC = 0.87, *P* < 0.001, 95% CI 0.75–0.99), and fall risk score higher than 10 points (AUC = 0.79, *P =* 0.002, 95% CI 0.65–0.92) were used for analysis in this study (Fig 1B).

### Uni- and multivariate logistic analysis

Univariate logistic regression analysis showed older age (>75 years), handgrip weakness, slower gait speed in the Get-up and Go test (>13.0 s), CES-D fatigue status, higher score of fall risk assessment (>10 points), and ASA score 3 were selected as preoperative significant factors, and rapid eGFR decline (>30%) was a postoperative risk factor for delirium. In multivariate analysis, slower gait speed in the Get-up and Go test (*P* = 0.008) and rapid eGFR decline (*P* = 0.003) were selected as an independent predictor for delirium (Table 3). Age (>75 years) showed marginal influences (*P* = 0.065) on delirium. Patients were categorized according to the number of independent predictors (scores 0–3). The occurrence of postoperative delirium was 0 for 122 (0%) patients with score 0 (= no risk factor), 1 for 68 (1.4%) patients with score 1 (= 1 risk factor), 5 for 19 (26%) patients with score 2 (2 risk factors), and 4 for 6 patients (67%) with score 3 (3 risk factors) (Fig 1C) This risk classification indicated significant association with the occurrence of postoperative delirium (*P <* 0.0001). To evaluate the predictive accuracy of the model, ROC curves were generated, and AUC was calculated. The AUC value in the risk classification was 0.95 (*P <* 0.001, 95% CI, 0.90–1.00; Fig 1D).

**Table 3:** Uni- and multivariate logistic regression analysis of independent risk factors for incident of postoperative delirium.

		Univariate analysis			Multivariate analysis		
	Preoperative risk factors	*P value*	OR	95%CI	*P value*	OR	95%CI
Age	> 75 yrs old	*0*.*002*	28.65	3.54–231	*0*.*065*	10.14	0.86–119
Gender	Male	*0*.*908*	0.91	0.19–4.46	*0*.*398*	2.60	0.28–24.0
Body mass index	Less than 20 kg/m^2^	*0*.*745*	1.30	0.26–6.41	*0*.*758*	1.51	0.11–21.2
Type 2 diabetes	Positive	*0*.*034*	4.74	1.12–20.0	*0*.*328*	2.84	0.35–22.9
Handgrip strength	Male <16kg,Female <18kg	*0*.*004*	6.81	1.83–25.4	*0*.*125*	4.10	0.67–25.0
Get-up and go	> 13 sec.	*0*.*000*	35.05	6.98–176	*0*.*008*	12.49	1.95–80.2
CES-D fatigue	Positive	*0*.*042*	4.45	1.06–18.72	*0*.*118*	4.80	0.67–34.3
Fall risk assessment	> 10	*0*.*005*	6.59	1.77–24.5	*0*.*916*	1.10	0.18–6.59
Nutritional status	GNRI < 92	*0*.*802*	0.156	0.156–11.0	*0*.*600*	2.81	0.06–133
ASA score	Score 3	*0*.*044*	3.89	1.04–14.6	*0*.*939*	1.07	0.18–6.25
		Univariate analysis			Multivariate analysis		
	Postoperative risk factors	*P value*	OR	95%CI	*P value*	OR	95%CI
Highest body temperature	> 38.0 °C	*0*.*609*	1.52	0.31–7.50	*0*.*660*	0.65	0.09–4.52
Clavien—Dindo grade	> 1	*0*.*713*	0.67	0.08–5.52	*0*.*581*	0.53	0.06–5.00
Types of surgery	Open	*0*.*053*	3.56	0.99–12.8	*0*.*215*	2.86	0.54–15.1
Anesthesia	General	*0*.*713*	1.48	0.18–12.2	*0*.*712*	0.64	0.06–6.75
Renal function decline	> 30% in eGFR	*0*.*002*	8.42	2.25–31.6	*0*.*003*	9.14	2.15–38.8
Serum albumin decline	> 30%	*0*.*167*	3.17	0.62–16.3	*0*.*602*	1.75	0.21–14.3
Blood loss	> 1000 g	*0*.*081*	4.41	0.83–23.3	*0*.*847*	1.29	0.10–17.5
Operation time	> 3 hours	*0*.*910*	0.92	0.23–3.68	*0*.*427*	0.50	0.09–2.76

## Discussion

In this study, the occurrence of postoperative delirium was 4.7% (10/215 patients). Older age (>75 years), slow gait speed (>13.0 s in Get-up and Go), and renal function decline (>30% decline in eGFR the day after surgery) were identified as risk factors for postoperative delirium. Although many studies have described rates and risk factors for postoperative delirium in major surgeries,[2, 5] only few reports have investigated rates and risk factors for postoperative delirium in urological surgery. Postoperative delirium incidences are reported to be 29% in radical cystectomy,[4] 21% in transurethral resection of prostate,[10] and 8.8% general urological surgery,[3] which were relatively higher than the present study. In those studies, delirium was screened and diagnosed using the Confusion Assessment Method, which may explain the higher incidence of delirium in comparison to the present study. Huge methodological differences among studies are important drawbacks to diagnose delirium. Therefore, the lack of acceptable explanation for the differences among various studies is an important limitation. Risk factors identified in those studies were older age (≥65 years), impairment in the instrumental activities of daily living, poor clock drawing test, geriatric depression scale, a previous history of delirium, and mental status examinations. However, all predictive factors cannot be applied into clinical practice without a well-trained geriatric care team, and therefore, an easy and simple method to evaluate frailty syndrome is required.

Recent systematic review demonstrated the predictive values of a slow gait speed in early death, disability, falls and hospitalization/institutionalization in the elderly (65 and older).[25] They recommended gait speed test for screening of sarcopenia in the elderly, which is a central element in the pathophysiology of frailty. The International Society of Geriatric Oncology (SIOG) also recommends the gait speed alone or included in composite tests, such as the timed Get-up and Go test and the Short Physical Performance Battery (SPPB) test, to assess functional status and mobility in a comprehensive geriatric assessment. [25] In addition, previous study suggested slow gait speed (the timed Get-up and Go test, >20 s) was significantly associated with the occurrence of early death within 6 months of first-line chemotherapy in heterogeneous elderly cancer population,[26] and postoperative delirium in major abdominal surgeries.[2] Therefore, gait speed is an important indicator of functional capacity and general frailty,[13] and it is positively correlated with cognitive frailty in elderly patients.[2, 27, 28] Based on these findings, we employed the timed Get-up and Go test as a predictor for frailty syndrome that could evaluate both gait speed and complex maneuvers. As expected, the results showed that slow gait speed (>13.0 s) was a strong predictor for postoperative delirium. This is the first report that identifies the cutoff value at 13 s in urological surgery. This may be caused by, at least in part, a study population difference between Western countries and Japan. Because definitive evidence regarding gait speed for delirium is yet to be established, a further larger scale study is required to address the issue of optimal cut-off values.

We first found that postoperative delirium is significantly frequent in nephrectomy or nephroureterectomy patients (in total, 60%, *P* = 0.003, Fisher’s exact test). Therefore, we hypothesized that the loss of rapid renal function may play an important role in the occurrence of postoperative delirium. We then investigated eGFR change between before and 1 day after surgery. The results indicated that rapid eGFR decline was significantly greater in the delirium group than that in the no-delirium group. In logistic regression analysis, eGFR decline > 30% was selected as a predictor for postoperative delirium. To our knowledge, this is the first report to identify the relationship between rapid renal function decline and postoperative delirium in urological surgery. Although our study could not address the background it can be hypothesized that the acute decline of renal function may induce an accumulation of anesthetizing agents, opioids, and/or metabolic substances after unilateral nephrectomy. Measuring the concentration of anesthetizing agent may help stratifying the risk for postoperative delirium. Further study is required to address these issues.

Next, we constructed a predictive model of postoperative delirium using independently significant factors. Because older age was reported as a strong predictor for postoperative delirium, we included older age (>75 years) for the predictive model of postoperative delirium. Based on these selected three factors, we stratified patients into four categories according to the number of independent predictors (scores 0–3). The occurrence of postoperative delirium was significantly higher in high score patients than that in lower score patients (*P* < 0.001). The AUC value of 0.95 indicated the high predictive accuracy of the risk stratification.

There are several limitations which should be reported. First, there is no data for cognitive measurement, including the Mini-mental Status Examination and lack of dementia diagnosis due to the lack of a well-trained geriatric care team at our hospital. We only used five delirium criteria to diagnose delirium. Based on five checklists, we were able to evaluate whether delirium was positive or negative, although not all nurses and doctors were well trained. However, because the investigators who diagnose delirium were not specialist for geriatric screening, it is difficult to ensure the quality of each assessment. Therefore, we could not exclude under detection of postoperative delirium, and systematically measure all types of delirium and severity.

Second, obvious demented people were excluded at the out-patient clinic because these patients were not indicated for urological surgery. The reason for the low delirium incidence in the present study might be the exclusion of demented people from surgery at out-patient clinic. The differences of indication of surgery among studies might be important drawbacks in incidence rates of delirium.

Third, the findings are not directly generalizable to other settings because of a single center study. The statistical power was inadequate for addressing the independent risk factors in this study due to the low occurrence of postoperative delirium. In addition, decision to set the cut-off value for the timed Get-up and Go test at 13.0 sec are not directly generalizable to other settings. Because no study assessed the implication of the timed Get-up and Go test in urological surgery and Japanese population, we statistically established our optimal cut-off value. Fourth, although we guaranteed that both the anesthetic management and the postoperative analgesia of patients were consistent, and it was not modified during this study, we could not address the unmeasurable confounders including anesthetic procedures, dosages of anesthetic agents, opioids, and dosages. Fifth, we did not assess the long-term complications of delirium. Because delirium is associated with medium and long-term poor outcomes, further investigations are necessary to assess the accuracy of delirium diagnosis and patients outcomes.

Despite these limitations, to our knowledge, this is the first report to investigate the relationship among gait speed, renal function decline, and occurrence of postoperative delirium. Our finding demonstrated that clinicians should be take special care in elderly patients with slow gait speed and uninephrectomy to address postoperative delirium. In addition, our data suggested that rapid renal function decline may have negative impact on the occurrence of postoperative delirium in other surgeries. Further validation studies that include cognitive measurement tools and other types of surgeries are warranted.

In conclusion, slow gait speed and rapid renal function decline after urological surgery independently associated with postoperative delirium. Although these factors must be validated in a larger cohort, our results will be helpful for perioperative patient management.

The minimal data set of the present study is available in S1 Dataset (Excel file).

## Supporting Information

S1 Dataset The minimal data set (Microsoft Excel file) of the present study. (XLSX)
